# Association of Emotional Self-Regulation with Psychological Distress and Positive Functioning Dimensions in Brazilian University Students during the COVID-19 Pandemic

**DOI:** 10.3390/ijerph20146428

**Published:** 2023-07-22

**Authors:** Maurício Rech, Gabriela Bertoletti Diaz, Bruno Luis Schaab, Carolina Garcia Soares Leães Rech, Prisla Ücker Calvetti, Caroline Tozzi Reppold

**Affiliations:** Research Laboratory for Psychological Assessment, Federal University of Health Sciences of Porto Alegre (UFCSPA), Porto Alegre 90050-170, Brazil; iciorech@hotmail.com (M.R.); gabrielabd@ufcspa.edu.br (G.B.D.); carolinagsleaes@gmail.com (C.G.S.L.R.); prisla@ufcspa.edu.br (P.Ü.C.); reppold@ufcspa.edu.br (C.T.R.)

**Keywords:** emotional regulation, COVID-19, university students, positive psychology, psychological distress

## Abstract

Emotional self-regulation is a relevant factor for human development capable of minimizing emotional difficulties in the face of adverse events, as was particularly useful during the COVID-19 pandemic. The present study aimed to evaluate emotional self-regulation in Brazilian health science university students and its relationship with positive psychology constructs (subjective well-being, hope, optimism, spirituality, self-compassion, and self-efficacy) and psychological distress (depression, anxiety, and stress). This was a prospective, cross-sectional, observational, analytic study of 1062 Brazilian undergraduate students with data collected using self-administered online questionnaires. Students in the first years of their undergraduate degree programs had significantly higher dysregulation scores than those in the final years. Multiple linear regression yielded a model that explained 71.8% of the variation in emotion dysregulation. The correlations of emotion dysregulation were significant and strong, scoring negatively with self-compassion, optimism, and subjective well-being and positively with psychological distress.

## 1. Introduction

In March 2020, the World Health Organization declared the coronavirus disease (COVID-19) a pandemic, along with a warning of the risk of increased levels of stress in the population. Major stressors during a pandemic include frustration and boredom due to the long quarantine period, virus or infection fears, loss of income, inadequate information, and stigma [1]. The abrupt and dramatic changes in everyday life associated with the systemic effects of the pandemic on the body, in particular on the brain and cognition, have a profound impact on the mental health of society in a broad context. Disruption in daily living routines can increase the degree of emotional damage to individuals and populations with pre-existing psychological symptoms or mental health disorders [2].

The mental health of university students was a matter of concern even before the pandemic, with evidence of a higher prevalence of anxiety and depressive symptoms in this population than in the general population [3,4], linked to characteristics related to transition, adaptation to the new environment, autonomy, social support, level of demands, and psychosocial stress [5]. Results even suggest heightened distress among university students in the field of health sciences [6,7,8]. In this setting, a Brazilian study of medical students showed increased incidence of depressive, anxiety, and stress symptoms [5].

The sudden emergence of the COVID-19 pandemic brought new elements to this set, with abrupt changes in social and learning routines, as students had to shift from face-to-face teaching to emergency remote online teaching, associated with adaptation difficulties and less positive emotional states. University students had significantly higher scores on depression, anxiety, and stress scales in online education during the pandemic than in classroom teaching [9,10].

In the context of a pandemic and in the face of a wide range of emotional difficulties, the positive psychology (PP) movement provides an important field of study concerned with and capable of promoting mental well-being, as it proposes a shift of focus from the pathology and remediation of suffering to the potentialities of positive individual characteristics [11]. In this respect, emotional self-regulation (ESR) [12] and other PP constructs appear as important factors that can minimize emotional difficulties and maladaptive behaviours, able to help people to overcome adversity during the time of COVID-19 pandemic [13].

ESR is defined as a process by which individuals develop the ability to influence how they deal with their own emotions, guided by conscious or unconscious strategies, and of being able to modify, inhibit, or activate their attention and behaviour based on previous experiences [14]. The construct is understood as an adequate way of dealing with experiences, characterized by the ability to successfully cope with aversive emotions and aiming at their regulation in stressful situations that may involve mood disorders [15]. The development of this ability is associated with intrinsic and extrinsic processes that include monitoring, evaluating, and modifying emotional reactions in the pursuit of predetermined goals. In the literature, coping strategies are divided into two groups: (a) adaptive strategies: those in which individuals, after defining goals and purposes based on personal values, increase their ability to recognize and process reactions that are useful to them, encouraging them to deal with situations more productively in the short and long term; and (b) non-adaptive or maladaptive strategies: those in which individuals manage to reduce the intensity of an emotion for a certain period of time, providing a momentary sense of well-being, but with a behaviour that does not match the goals and purposes of which they would approve [12]. The use of adaptive coping strategies is often associated with more successful academic performance and more positive social outcomes. Conversely, the use of maladaptive strategies can have a significant negative impact on an individual’s mental health [16].

Investigations of subjective well-being, optimism, hope, spirituality, self-efficacy in higher education, and self-compassion have associated these constructs either positively with ESR or negatively with emotion dysregulation. In recent decades, studies have empirically demonstrated that these PP constructs increase the human capacity to deal with emotional difficulties and can improve the mental health of the general population, including university students [12,17,18]. Regarding the presence of emotional and mental health difficulties, different studies have shown a negative correlation of ESR with anxiety, depression, and stress [14,15]. During the COVID-19 pandemic, publications pointed to the same conclusion: that the use of different ESR strategies can reduce distress [19,20,21].

In view of the foregoing, considering times of adversity such as the COVID-19 pandemic and their impacts on the mental health of university students, ESR and other PP constructs are important psychological resources [22]. University students in the field of health sciences who come into close contact with health care services are particularly at risk of emotional difficulties given the very object of their studies, in intellectual and practical terms, which exposes them to risks and increases their susceptibility to the development of psychological distress and mental disorders [23].

The variables chosen for this study in the field of positive psychology are emotional self-regulation, subjective well-being, self-compassion, spirituality, optimism, hope, and self-efficacy. These variables have been extensively researched in the domain of positive mental health worldwide and appear to function as protective factors for the emotional well-being of diverse populations [24,25]. Moreover, there are validated and reliable instruments available in Brazil for measuring these constructs [26,27].

Thus, the present study aimed to evaluate ESR in Brazilian undergraduate health science students and (a) its relationship with PP constructs, specifically subjective well-being, spirituality, optimism, hope, self-compassion, and self-efficacy in higher education, as well as (b) its relationship with psychological distress (depression, anxiety, and stress scores). In this vein, as the first hypothesis of the present study, we expected to find weak-to-strong significant positive correlations between ESR and PP constructs—namely, subjective well-being, optimism, hope, spirituality, self-compassion, and self-efficacy in higher education—or, conversely, negative correlations between emotion dysregulation and the same PP constructs. Therefore, as the second hypothesis of the present study, we expected to find a negative correlation between ESR and distress or, conversely, a moderate-to-strong positive correlation between emotion dysregulation and distress in Brazilian undergraduate health science students during the COVID-19 pandemic.

## 2. Materials and Methods

### 2.1. Sample

The convenience sample consisted of 1191 university students from all 5 geographic regions of Brazil (M = 24.69; SD = 7.60). Of these, 1062 (89.17%) were included in the study because they were undergraduate students in the field of health sciences, whereas 129 (10.83%) were not eligible for inclusion either because they were attending courses in fields other than health sciences (10.50%) or for not signing the informed consent form (0.33%).

### 2.2. Instruments

Psychometric instruments were selected based on their previous validation in Brazilian studies. Additionally, they should provide appropriate precision data. The following instruments were used for data collection.

#### 2.2.1. Sociodemographic Questionnaire

An electronic questionnaire was designed to collect data on participants’ gender, age, region of the country where they lived, whether they were attending a public or private university, stage of course (first or final years), class shift, and whether they were working during the pandemic.

#### 2.2.2. Emotional Dysregulation Scale—Adults—EDEA

Based on the theoretical assumptions of Gratz and Roemer (2004) [14], this short version of the scale was developed for the Brazilian context by Cremasco et al. (2020) [28]. The EDEA is a 15-item, self-report scale designed to evaluate 4 factors. Factor 1 consists of 4 items that assess appropriate coping strategies. Factor 2 consists of 3 items that assess the externalization of aggression. Factor 3 consists of 4 items that assess pessimism. Factor 4 consists of 4 items that assess paralysis. Respondents rate on a 4-point Likert scale how well their behaviours, feelings, and thoughts describe them in the face of sad events. Factor 1 items are reverse scored. Higher total scores indicate greater emotion dysregulation and, therefore, lower ESR. The scale has adequate internal consistency (Cronbach α = 0.94), with internal structure evidence of validity and data normalized for the Brazilian population. In the present study, the scale also had adequate internal consistency, with Cronbach α = 0.88.

#### 2.2.3. Positive and Negative Affect Schedule—PANAS

Developed by Watson and Clark (1994) [29], this scale had its current rules of score interpretation presented by Zanon and Hutz [30]. PANAS is a 20-item, self-report scale, with 10 items assessing positive affects (PA) and 10 items assessing negative affects (NA). The scale consists of adjectives for which respondents indicate a number that corresponds to what extent they have felt the emotion described by the adjective. The answers are recorded on a 5-point Likert scale. The scale has adequate internal consistency (Cronbach α = 0.83 for PA; α  =  0.77 for NA), with internal structure evidence of validity and data normalized for the Brazilian population. In the present study, the scale also had adequate internal consistency, with Cronbach α = 0.88 (PA) and α = 0.89 (NA).

#### 2.2.4. Satisfaction with Life Scale—SWLS

Originally developed by Diener et al. (1985) [31], the Brazilian version of the scale was adapted and validated by Zanon et al. (2013) [32]. It is a 5-item, self-report scale designed to assess a person’s level of satisfaction with life as a whole. The answers are recorded on a 7-point Likert scale in which respondents indicate a number that corresponds to how much they agree or disagree with the statements. The scale is composed of a single factor and has high internal consistency (α = 0.87) and high test–retest reliability (r  =  0.82). It has internal structure evidence of validity and data normalized for the Brazilian population. In the present study, the scale also had adequate internal consistency, with Cronbach α = 0.85.

#### 2.2.5. Spirituality Self-Rating Scale—SSRS

Originally developed by Galanter et al. (2007) [33], the Brazilian version of the scale was adapted and validated by Gonçalves and Pillon (2009) [34]. SSRS is a 6-item, self-report scale designed to assess 3 factors: peace, meaning, and faith. The scale assesses one’s spirituality by asking participants to rate on a 5-point Likert scale the importance of spiritual orientation and how they apply it to their lives. It has adequate internal consistency (Cronbach α = 0.83), with internal structure evidence of validity and data normalized for the Brazilian population. In the present study, the scale also had adequate internal consistency, with Cronbach α = 0.91.

#### 2.2.6. Revised Life Orientation Test—LOT-R

The scale was developed by Scheier and Carver (1985) [35] to measure dispositional optimism, and the Brazilian version was adapted by Bastianello et al. (2014) [36]. It is a self-report test consisting of 10 items, 3 of which are statements about optimism and 3 about pessimism, and 4 filler items whose scores are not considered. Respondents are asked to indicate the extent to which they agree with each of the items using a 5-point Likert scale. Like the original instrument, the validated and standardized instrument used in Brazil is unidimensional and has adequate internal consistency (Cronbach α = 0.80). In the present study, the scale also had adequate internal consistency, with Cronbach α = 0.857.

#### 2.2.7. Cognitive Hope Scale

Developed based on the Hope Index by Staats (1989) [37], the Brazilian version has 5 additional items in relation to the original scale, according to previous studies on the content-related evidence of validity in the Brazilian context. The scale consists of 21 items that measure self-centred hope and altruistic hope. It has 2 columns, each representing a subscale: desire (how much you want something) and expectation (how much you believe that it will happen). Participants should be informed in advance that they must give 2 answers for each item, one for each column, along a Likert scale to evaluate each model of hope (self-centred and altruistic). The cognitive hope score is calculated by multiplying the scores indicated for each item in the desire and expectation columns, and the sum of the results determines the global cognitive hope score. The scale has internal structure evidence of validity, with Cronbach α = 0.86 for self-centred hope and α = 0.80 for altruistic hope, and data normalized for the Brazilian population [38]. In the present study, the scale also had adequate internal consistency for cognitive hope, with Cronbach α = 0.91.

#### 2.2.8. Self-Compassion Scale—SCS

Originally developed by Neff (2003) [39], the scale was adapted to the Brazilian context by Souza and Hutz (2016) [40]. It is a 26-item, self-report scale divided into 6 factors. The self-kindness and self-judgment factors are assessed separately, with 5 items each, and the common humanity, isolation, mindfulness, and over-identification factors are also assessed separately, with 4 items each. Respondents are asked to indicate how often they have acted in the manner stated in each of the items on a 5-point Likert scale. The total score is the sum of all responses, with the scores for the self-judgment, isolation, and over-identification factors being reverse scored. Therefore, higher total SCS scores indicate higher levels of self-compassion. The scale was validated and standardized for use in Brazil, with adequate internal consistency (Cronbach α = 0.92). In the present study, the scale also had adequate internal consistency, with Cronbach α = 0.94.

#### 2.2.9. Higher Education Self-Efficacy Scale

This 34-item, self-report scale was developed based on the Guide for Constructing Self-Efficacy Scales by Bandura (2006) [41]. The answers are recorded on a 10-point Likert scale in which respondents indicate how confident they are that they can perform a given task, with the aim of assessing 5 factors: academic self-efficacy, academic self-regulatory efficacy, proactive self-efficacy, social self-efficacy, and self-efficacy for self-regulated learning. The validated scale has adequate internal consistency (Cronbach α = 0.94) [42]. In the present study, the scale also had adequate internal consistency, with Cronbach α = 0.96.

#### 2.2.10. Depression Anxiety Stress Scales—DASS-21

Originally developed by Lovibond and Lovibond (1995) [43], the short version (DASS-21) validated in Brazil was reconciled by Martins et al. (2019) [44] with minor cultural adaptations. It is a 21-item, self-report scale that contains 3 subscales, each of which consists of 7 items that assess the states of depression, anxiety, and stress. The answers are recorded on a 4-point Likert scale. The internal consistency of the scale was indicative of adequate reliability, as estimated by using composite reliability (CR) and an ordinal alpha coefficient (α), with values ≥ 0.70 for both CR and α. In the present study, unidimensional analysis with data correction was the best model to measure general distress [45], yielding adequate internal consistency (Cronbach α = 0.94).

### 2.3. Procedures

This was a prospective, cross-sectional, observational, analytic study with data collected at a single time point using an online questionnaire (Google Forms). The survey was released as follows: (1) by email, we contacted public and private Brazilian universities (mentors and academic deans in undergraduate programs), who forwarded the survey link to students in the field of health sciences in their institutions; and (2) through social media. Data were collected from 24 May to 1 August 2021. Initially, potential participants were provided with the consent form, which explained the research objectives and the ethical considerations associated with it. Subsequently, individuals who agreed to participate accessed the sociodemographic questionnaire, along with the positive psychology psychometric instruments. Participation was voluntary, and on average, participants spent approximately 25 min completing all the questions. The survey was extensively promoted in Brazil, ensuring a robust sample. The inclusion of a complete sample from the health field, along with data collection during the remote teaching period, probably contributed to the sample size of this research.

### 2.4. Ethical Considerations

The study was approved by the institution’s research ethics committee and conducted in accordance with ethical guidelines for research involving human subjects. Participation was voluntary, and anonymity was preserved. Written informed consent was obtained from each participant prior to inclusion in the study. All procedures complied with the current guidelines for research in virtual environments.

### 2.5. Statistical Analysis

The results of qualitative variables were expressed as frequency and percentage, and of quantitative variables as mean and standard deviation. The normality of data was assessed by visual inspection of histograms. The sample was characterized according to gender, where the association with qualitative variables was assessed by the chi-square test with adjusted standardized residual analysis, and the association with quantitative variables by Student’s *t* test; the gender “Other” was not analysed due to the low rate (1.1%). Pearson’s correlation coefficient was used to assess correlations between the components of the emotion dysregulation scale and of these components with the other scales and age. Student’s *t* test and analysis of variance (ANOVA) were used to compare emotion dysregulation scores with qualitative variables, and Tukey’s test was used for multiple comparisons.

Four multiple linear regression models were estimated to explain dysregulation using qualitative variables and the scales that showed significant associations/correlations: (a) using subjective well-being, without PA, NA, and life satisfaction, with the 3-factor DASS; (b) using PA, NA, and life satisfaction, without subjective well-being, with the 3-factor DASS; (c) using subjective well-being, without PA, NA, and life satisfaction, with the 1-factor DASS; and (d) using PA, NA, and life satisfaction, without subjective well-being, with the 1-factor DASS. While the study was primarily conceptual in nature, it was found that certain sociodemographic variables, when interacting with positive psychology variables and distress, provided a more comprehensive representation of emotion dysregulation scores. As a result, it was decided to incorporate these variables into the models. Dummy variables were used to include sociodemographic data in the models [46]. Since the recommended sample size for multiple linear regressions is 15 participants per predictor variable [46], it can be concluded that the study had a sufficient sample size.

The absence of multicollinearity was determined by a variance inflation factor < 5. Residuals were checked for the following assumptions: normality (visual inspection of histograms), absence of autocorrelation (Durbin–Watson statistic close to 2), and homoscedasticity (scatter plot of predicted × unstandardized residuals). A forward stepwise selection procedure was used. Results with *p* < 0.05 were deemed statistically significant. Data analysis was conducted using SPSS, version 25.0 (IBM-SPSS Statistics for Windows; IBM Corp., Armonk, NY, USA).

## 3. Results

The general characteristics of the sample are summarized in Table 1.

The emotion dysregulation scale scores were significantly higher in women than in men. The dysregulation scores differed significantly between the regions of the country, with higher scores in the Southeast than in the South. Students attending public universities had significantly higher dysregulation scores than those attending private universities. Students in the first years of their undergraduate degree programs had significantly higher dysregulation scores than those in the final years. Table 2 shows the relationships of ESR scores according to sample characteristics.

Four multiple linear regression models were estimated, and all of them could explain about 70% of the variation in dysregulation (adjusted R² values were about 0.7). The model that best explained the variation in emotion dysregulation, as a dependent variable, consisted of the following variables: self-compassion, NA, distress, optimism, age, gender, and higher education self-efficacy. This model was significant (F = 386.3; gl: 7 and 1054) and explained 71.8% (adjusted R² = 0.718) of the variance in emotion dysregulation scores. In this model, self-compassion, optimism, age, and higher education self-efficacy were associated with significantly lower emotion dysregulation scores, whereas NA, distress, and female gender were associated with significantly higher dysregulation scores. Participants had a mean decrease in emotion dysregulation of 5.13 points for each additional point in self-compassion, of 0.17 points for each additional point in optimism, of 0.41 points for each additional point in higher education self-efficacy, and of 0.08 points for each additional year of age. Participants had a mean increase in emotion dysregulation of 0.23 points for each additional point in NA and of 0.10 points for each additional point in distress. The female gender showed a mean increase of 1.38 points in emotion dysregulation compared with the male gender. Table 3 shows the multiple linear regression model that best explained the variation in emotion dysregulation.

All six PP constructs evaluated here had a weak-to-strong negative correlation with emotion dysregulation, ranging from –0.18 to –0.78. Strong correlations were observed for self-compassion, optimism, and subjective well-being; moderate correlations for cognitive hope and higher education self-efficacy; and a weak correlation for spirituality. A significantly strong positive correlation was found between emotion dysregulation and distress. Table 4 shows all the correlations between emotion dysregulation, PP constructs, and distress.

## 4. Discussion

The found differences in emotion dysregulation among audience groups are aligned with findings from the existing literature on mental health. Individuals from the Southeast region of Brazil reported higher levels of self-reported depression and anxiety [47,48]. Therefore, it is reasonable to expect that emotional dysregulation would be more pronounced among this population, given the relation between these variables. Previous research also indicated that women may tend to present more difficulties in emotional self-regulation compared to their male counterparts [49], and to experience greater psychological distress [47,50], which supports this result.

Income represents a potential explanation for the heightened emotion dysregulation observed among students attending public universities. A previous study has suggested that income is a risk factor for mental health issues during periods of social isolation [51]. Moreover, students in the early stages of their undergraduate studies displayed increased levels of emotion dysregulation. This may be partially attributed to the developmental period they are undergoing, as adolescence and the transitional phase into adulthood are commonly associated with heightened emotional impairments [52]. The challenges posed by the pandemic may have further exacerbated these conflicts.

The results of the present study confirmed both the general hypothesis of weak-to-strong significant correlations between ESR and the PP constructs studied, and the specific hypothesis of a negative correlation between ESR and distress. Because the EDEA scale [28] was used in the present study, it is important to point out that the scores should be reversed for interpretation and description of ESR results, where high levels of emotion dysregulation are proportional to low levels of ESR and, conversely, low levels of emotion dysregulation are proportional to high levels of ESR.

As expected, a strong negative correlation was found between emotion dysregulation scores and optimism. This result is supported by a recent study of 366 university students in the field of health sciences conducted in Tunisia that evaluated students’ mental health during the COVID-19 pandemic [19]. The results showed a positive correlation between ESR and optimism (β: 0.33, SE: 0.06, *p* < 0.05), which explained 11% of the total variance between them, further demonstrating that higher levels of emotion regulation can lead to a more optimistic view of life. Optimism is a trait that contributes to mental well-being, thus strategies for fostering optimism have been employed as an emotional self-regulation technique in positive psychotherapy [25].

The present study showed a strong negative correlation between emotion dysregulation and self-compassion. This result is supported by an Australian study with 750 undergraduate students that obtained a positive association between ESR and self-compassion, specifically demonstrating that emotion regulation is a mechanism that influences the impact of self-compassion on social anxiety [53]. A systematic review identified studies that found negative associations between self-compassion and dysfunctional emotion regulation. The results demonstrated that emotion dysregulation mediated the relationship of self-compassion with depression and post-traumatic stress disorder symptoms. Conversely, self-compassion mediated the relationship between dysfunctional emotion regulation and post-traumatic stress disorder [54]. The association between self-compassion and emotional self-regulation is firmly established, leading to the integration of self-compassion as a core component in various psychotherapeutic approaches [55]. Compassion-Focused Therapy and Mindful Self-Compassion are examples of theoretical frameworks that employ self-compassionate exercises to facilitate emotional regulation and, consequently, enhance mental well-being [56].

The present study confirmed the strong negative correlation of subjective well-being scores with emotion dysregulation scores. Our results are consistent with recent studies of university students demonstrating a positive association of subjective well-being with adaptive emotion regulation strategies and a negative association with maladaptive strategies. A study of 84 psychology students in Argentina observed that adaptive cognitive strategies for emotion regulation were positively related to subjective well-being, whereas maladaptive strategies had a significant negative relationship with this construct [57]. Likewise, a study of 350 university students in Pakistan revealed that emotion regulation, using both adaptive and maladaptive strategies, had a significant direct effect on subjective well-being in young adults [58]. Given that the primary goal of psychotherapeutic approaches is the cultivation of emotion regulation skills, it is anticipated that individuals with lower emotion regulation abilities will exhibit higher levels of psychological distress [55]. Conversely, individuals who possess greater resources for emotion regulation, such as effective coping strategies, tend to demonstrate a more favourable mental health status [59].

The present study also found a moderate negative correlation between emotion dysregulation scores and cognitive hope scores. This association is supported by a study of 233 university students in Brazil demonstrating a significant correlation between the character strength of hope and all four factors of ESR [12]. The study found significantly higher moderate correlations for coping strategies (r = 0.33) and pessimism (r = −0.33), and weak correlations for externalization of aggression (r = −0.18) and paralysis (r = −0.28). Our findings are consistent with these results, as they associated hope positively with adaptive emotion regulation strategies and negatively with maladaptive strategies. Cognitive hope may also serve as an effective emotional self-regulation strategy, aiding in the management of distressing situations. Engaging in hope-related exercises during traumatic moments, such as when experiencing illness, can contribute to emotional well-being throughout the course of these experiences [25].

Moderate negative correlations were also found between emotion dysregulation scores and higher education self-efficacy scores. To date, no study has specifically correlated self-efficacy in higher education with ESR. However, an international study on university students has found positive correlation of academic self-efficacy with emotion regulation [60]. In addition to being consistent with the results of the aforementioned study, the present findings are also supported by the very definition of self-efficacy, understood as individuals’ confidence that they can perform a given task based on their own cognitive, motivational, and behavioural resources.

Spirituality was the only construct to show a weak negative correlation with emotion dysregulation scores. The hypothesis of an association between ESR and spirituality has already been demonstrated in the literature [17]. However, a recent study of university students did not find such a significant direct relationship [12]. Although spirituality and religiosity are overlapping constructs, they are nonetheless distinct, and only a few studies have contrasted these two pathways, especially in relation to emotion regulation [17]. Similarly to the aforementioned constructs, spirituality can also play a role in mental health [61]. Individuals with a higher level of spirituality may possess cognitive coping strategies that attenuate the impact of negative emotions, such as holding the belief that a religious figure cares for and safeguards them.

In addition to the negative correlations with PP constructs, strong positive correlations were also found between emotion dysregulation and distress. These data are supported by a recent study of university health science students in Tunisia (*n* = 366), with results showing a significant negative relationship between emotion regulation and distress (stress = β: −0.47, SE: 0.06, *p* < 0.05; anxiety = β: −0.30, SE: 0.07, *p* < 0.05; depression = β: −0.26, SE: 0.07, *p* < 0.05), further highlighting the potential protective role of emotion regulation for students’ mental health during the spread of the COVID-19 pandemic [19]. In another recent study of 620 undergraduate students in Turkey, distress was significantly and positively associated with difficulties in emotion regulation [20]. All these results are in line with different pre-pandemic studies, which point to the negative correlation of ESR with anxiety, depression, and stress [15,62]. Given that emotional self-regulation is a focal skill in psychotherapeutic approaches, it is anticipated that university students with lower levels of emotional self-regulation would experience greater psychological distress. Positive psychotherapy, which incorporates emotional self-regulation strategies, draws upon constructs examined in this research, including optimism, hope, spirituality, and self-compassion [25].

## 5. Conclusions

The theoretical contribution of the study is that it highlights the influence of PP constructs on emotional self-regulation in a large sample size, representative of students from different undergraduate degree programs in the field of health sciences across all five regions of the country. Regarding limitations, although appropriate statistical methods were used to ensure the reliability of the results, our study was cross-sectional and based on self-report questionnaires, with a predominantly female sample. In addition, most of the sample came from the South and Southeast regions. Finally, it is important to acknowledge that mental health status cannot be solely attributed to the effects of COVID-19, as the data obtained from the current sample were not controlled for the pre-pandemic period.

In conclusion, our results indicate that self-compassion, optimism, and subjective well-being are strongly correlated with ESR, and that higher levels of emotion dysregulation are associated with heightened distress and decreased mental health. These findings corroborate the two hypotheses of the study and reveal the importance of emotional self-regulation for the mental health of college students.

Further intervention studies with strategies to promote PP constructs are warranted to assess their impact on students’ mental health by measuring levels of emotion dysregulation, distress, and self-efficacy scores in higher education. Given the link between ESR and key constructs within positive psychology, as well as psychological distress, it is imperative to encourage further research aimed at developing effective strategies for enhancing emotional self-regulation. Interventions centred around self-compassion, for instance, hold promise in fostering the emotional well-being of university students, both during and in the aftermath of the COVID-19 pandemic.

## Figures and Tables

**Table 1:** General characteristics of the sample of Brazilian undergraduate health science students (*n*  = 1062).

Characteristic	*n*	%
Gender		
Female	837	78.8
Male	213	20.1
Other	12	1.1
Region of the country		
South	481	45.3
Southeast	402	37.9
Midwest	35	3.3
Northeast	123	11.6
North	21	2.0
University		
Public	847	79.8
Private	215	20.2
Stage of course		
First years (1st–4th semester)	450	42.4
Final years (5th–12th semester)	612	57.6
Class shift		
Mixed shifts	698	65.7
Single shift	364	34.3

**Table 2:** Relationships of emotional self-regulation scores according to sample characteristics.

Characteristic	Self-Regulation Score		*p*-Value
	Mean	SD	
Gender			0.000
Female	21.85	8.83	
Male	18.46	8.99	
Region of the country			0.006
South	20.26	8.66	
Southeast	22.47	8.77	
Midwest	22.34	9.94	
Northeast	20.56	9.89	
North	20.95	9.56	
University			0.001
Public	21.68	8.98	
Private	19.39	8.64	
Stage of course			0.015
First years (1st–4th semester)	21.99	8.85	
Final years (5th–12th semester)	20.64	9.00	
Class shift			0.066
Mixed shifts	21.58	9.05	
Single shift	20.51	8.76	

SD: standard deviation.

**Table 3:** Linear regression model that explains 71.8% of the variation in emotion dysregulation.

Model	Unstandardized Coefficients		Standardized Coefficients	t	*p*-Value	95% CI for β
	β	Standard Error	β			
(Constant)	32.96	1.47		22.49	0.000	30.09 to 35.84
Self-compassion	−5.13	0.27	−0.45	18.83	0.000	−5.66 to −4.59
Negative affects	0.23	0.02	0.23	9.97	0.000	0.19 to 0.28
Distress	0.10	0.02	0.15	6.32	0.000	0.07 to 0.13
Optimism	−0.17	0.04	−0.11	−4.67	0.000	−0.24 to −0.10
Age	−0.08	0.02	−0.06	−3.83	0.000	−0.12 to −0.04
Higher education self-efficacy	−0.41	0.11	−0.07	−3.76	0.000	−0.63 to −0.20
Gender (female)	1.15	0.37	0.05	3.11	0.002	0.42 to 1.87

95% CI: 95% confidence interval.

**Table 4:** Correlations between emotion dysregulation and positive psychology constructs.

Pearson’s Correlation	r
Spirituality score	−0.177 *
Optimism score	−0.646 *
Self-kindness	−0.598 *
Self-judgment	0.591 *
Common humanity	−0.485 *
Isolation	0.695 *
Mindfulness	−0.623 *
Over-identification	0.723 *
Self-compassion score	−0.778 *
Academic self-efficacy	−0.372 *
Academic self-regulatory efficacy	−0.363 *
Proactive self-efficacy	−0.312 *
Social self-efficacy	−0.357 *
Self-efficacy for self-regulated learning	−0.300 *
Higher education self-efficacy score	−0.390 *
Altruistic hope	−0.264 *
Self-centred hope	−0.457 *
Cognitive hope score	−0.441 *
Depression	0.675 *
Anxiety	0.482 *
Stress	0.592 *
Subjective well-being score	−0.641 *
Negative affects	0.665 *
Positive affects	−0.417 *
Life satisfaction score	−0.443 *
Distress	0.663 *

* *p* < 0.0001.

## Data Availability

The data that support the findings of this study are available from the corresponding author, B.L.S., upon reasonable request.
